# Supplementary material for: Impact of Extended Use of Ablation Techniques in Cirrhotic Patients with Hepatocellular Carcinoma: A Cost-Effectiveness Analysis
Source: Cancers (Basel). 2022 May 26;14(11):2634. doi: 10.3390/cancers14112634 (PMC9179352; doi:10.3390/cancers14112634)
Supplement: Supplementary file 1 [file cancers-14-02634-s001.zip › cancers-1737058-supplementary.pdf]

SUPPLEMENTARY MATERIAL

Impact of extended use of ablation techniques in cirrhotic patients with hepatocellular carcinoma: a cost-effectiveness analysis

Table of Contents

Markov Model

Figure S1. Simplified state transition model illustrating the progression of the disease and possible interventions……………………...4

Figure S2. Markov model of cirrhotic patient with HCC treated by hepatic resection in first-line strategy……………………..5

Figure S3. Markov model of cirrhotic patient with HCC treated by ablation in first-line strategy……………………..6

Figure S4. Markov model of cirrhotic patient with HCC treated by TACE in first-line strategy……………………..7

Figure S5. Markov model of cirrhotic patient with HCC treated by Sorafenib in first-line strategy……………………..8

Table S1. Transition probabilities of cirrhotic patient with HCC treated by hepatic resection in first-line strategy……………………...9

Table S2. Transition probabilities of cirrhotic patient with HCC treated by ablation in first-line strategy……………………..10

Table S3. Transition probabilities of cirrhotic patient with HCC treated by TACE in first-line strategy……………………..11

Table S4. Transition probabilities of cirrhotic patient with HCC treated by Sorafenib in first-line strategy……………………..12

Figure S6. Model calibration by first-line treatments in the (A) Bondy and the (B) SOC group……………………..13

Propensity score Analysis

Table S5. Characteristics of variables including in the model used for propensity score……..15

Figure S7. Balance assessment on the covariates after propensity score matching………...16

Costs Evaluation

Table S6. Costs of in-patient HCC treatment from PMSI………………………...….17

Table S7. Costs of pre-operative examinations……………………………...…….18

Table S8. Direct medical costs of out-patient HCC treatment…………………...…..19

Table S9. Costs of follow-up surveillance after curative or palliative treatment.………...20

Table S10. Costs of in-patient follow-up (care after liver transplantation, palliative care) from PMSI...21

Table S11. Direct medical costs of HCC management from French Insurance..22

Table S12. Results of the cost-effectiveness analyses, by first-line treatment on a 5-year horizon...23

Figure S8. Results of the deterministic univariate analysis……………………...…..24

Markov Model

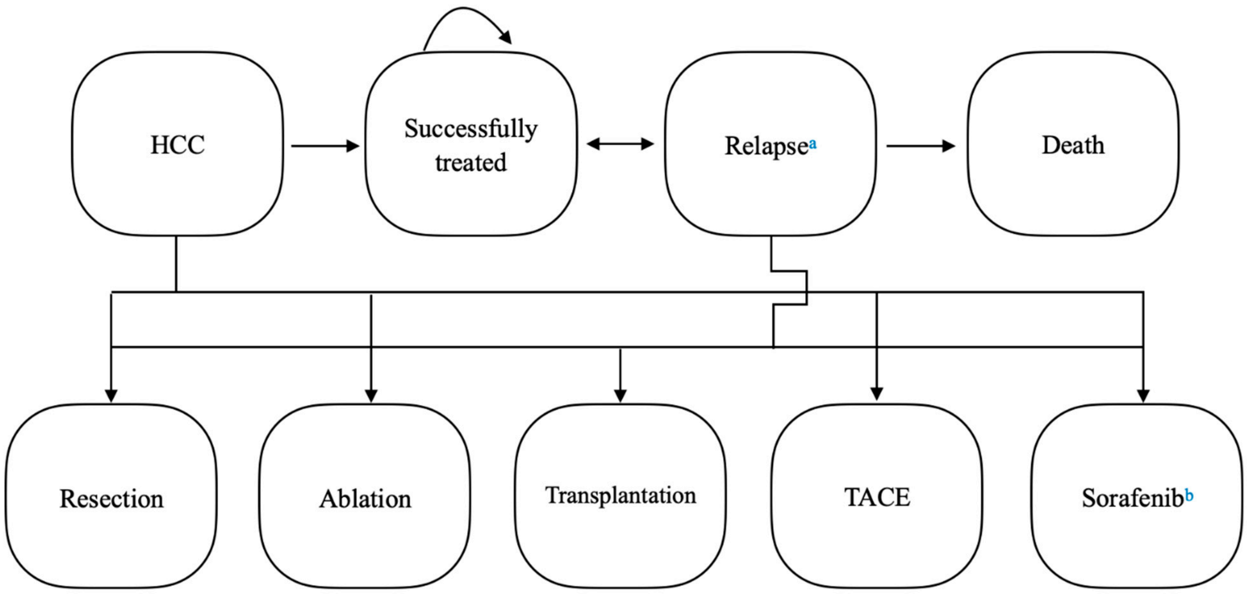

Figure S1. Simplified state transition model illustrating the progression of the disease and possible interventions.

[a]Recurrence refers to development of new HCC lesions, relapse at the site of previous treatment, or non-response, necessitating repeated treatment at least 3 months after the previous one or switching to another treatment modality.

[b]TARE and Sorafenib are combined into one group. HCC, Hepatocellular carcinoma; TACE, trans-arterial chemoembolization; TARE, trans-arterial radioembolization.

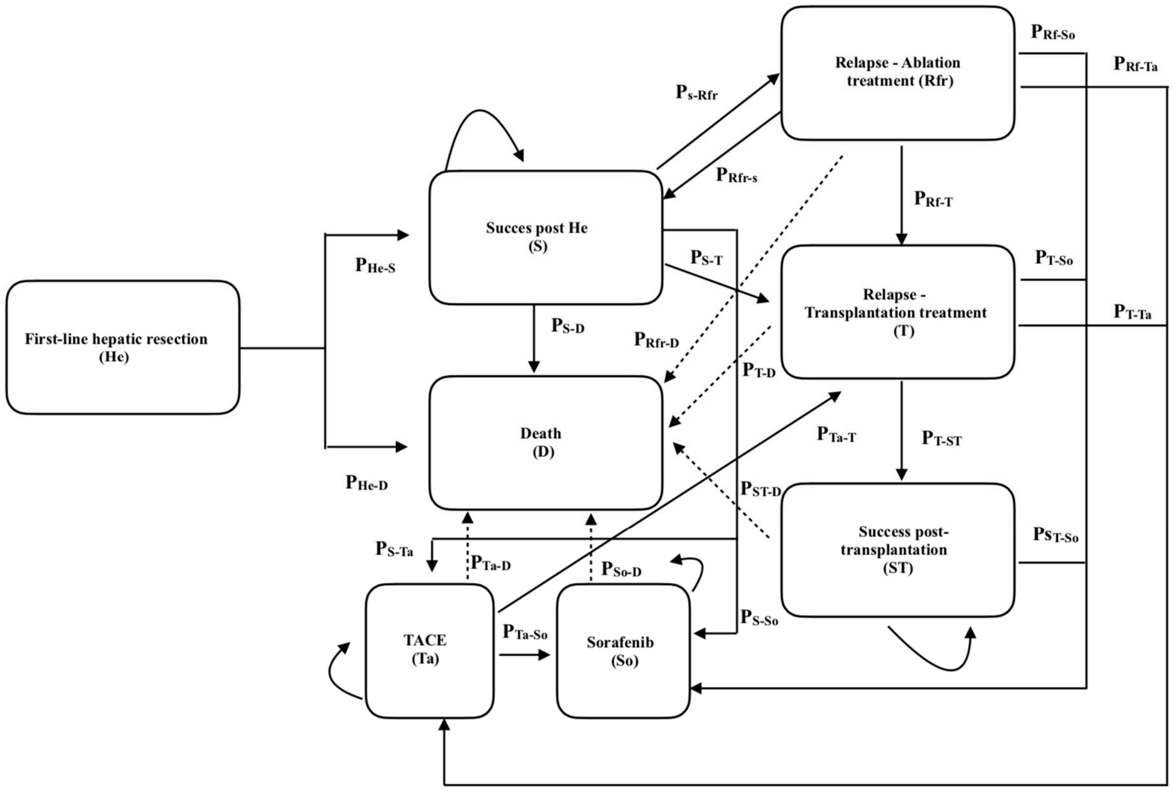

Figure S2. Markov model of cirrhotic patient with HCC treated by hepatic resection in first-line strategy

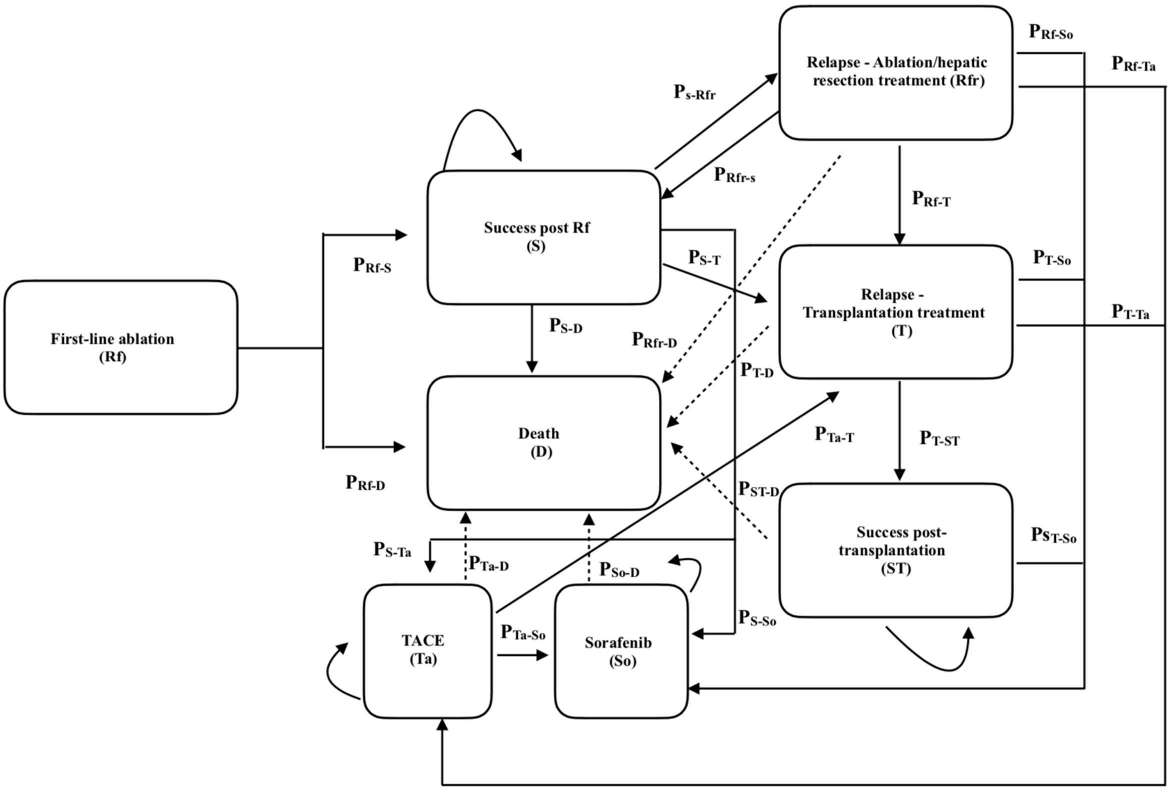

Figure S3. Markov model of cirrhotic patient with HCC treated by ablation in first-line strategy.

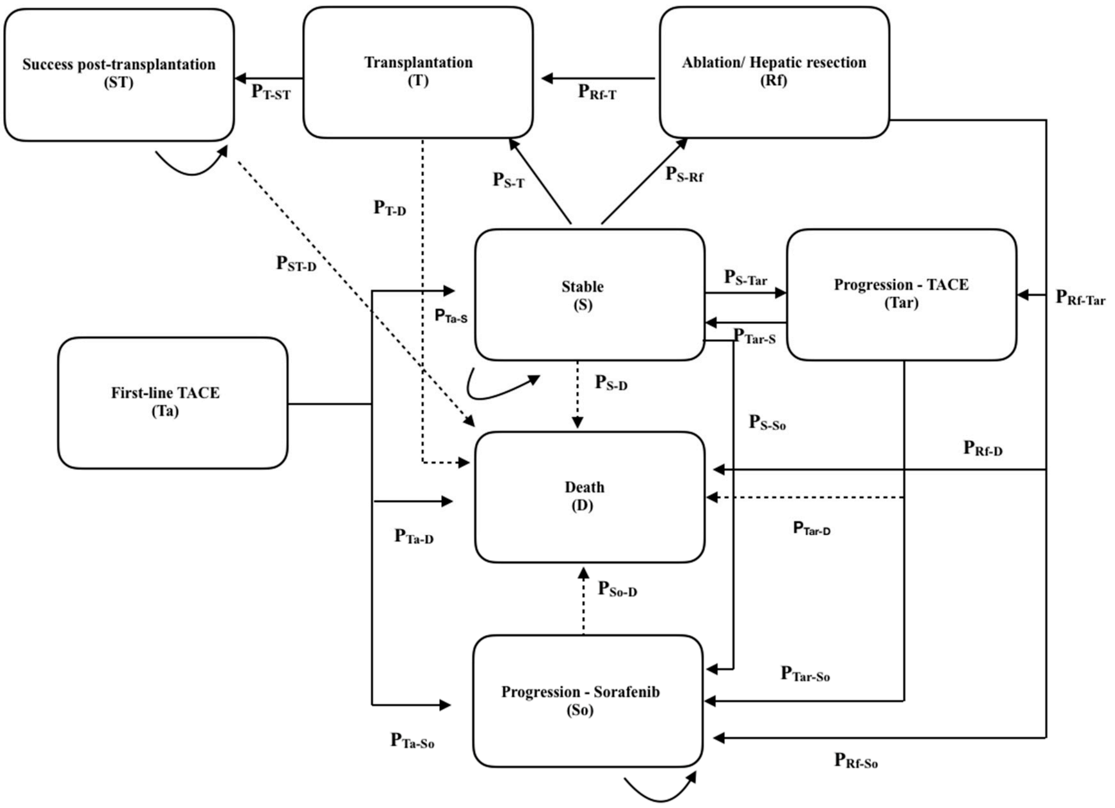

Figure S4. Markov model of cirrhotic patient with HCC treated by TACE in first-line strategy.

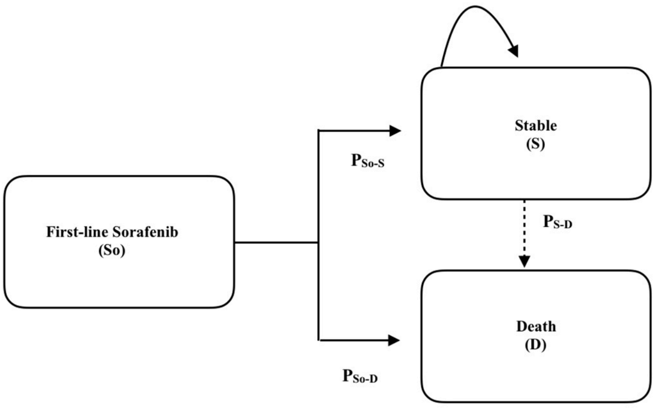

Figure S5. Markov model of cirrhotic patient with HCC treated by Sorafenib in first-line strategy

Table S1. Transition probabilities of cirrhotic patient with HCC treated by hepatic resection in first-line strategy

Transition probabilities	Bondy group	SOC group
p(He-D)	0.00%	0.00%
p(S-Rfr)	4.73%	4.73%
p(S-T)	0.00%	0.00%
p(S-So)	0.00%	0.00%
p(S-D)	2.99%	2.99%
p(S-Ta)	0.00%	0.00%
p(Rfr-T)	14.13%	14.13%
p(Ta-T)	0.00%	0.00%
p(ST-So)	0.00%	0.00%
p(ST-D)	0.00%	0.00%
p(Rfr-D)	0.00%	0.00%
p(T-D)	0.00%	0.00%
p(Rfr-So)	0.00%	0.00%
p(T-So)	0.00%	0.00%
p(Rfr-Ta)	0.00%	0.00%
p(T-Ta)	0.00%	0.00%
p(So-D)	0.00%	0.00%
p(STa-D)	4.73%	4.73%
p(STa-Ta)	0.00%	0.00%
P(STa-So)	0.00%	0.00%
p(Ta-So)	2.99%	2.99%
p(Ta-D)	0.00%	0.00%

Table S2. Transition probabilities of cirrhotic patient with HCC treated by ablation in first-line strategy

Transition probabilities	Bondy group	SOC group
p(Rf-D)	1.11%	0.93%
p(S-Rfr)	12.89%	12.60%
p(S-T)	0.73%	0.61%
p(S-So)	1.25%	1.52%
p(S-D)	4.97%	4.50%
p(S-Ta)	2.05%	2.01%
p(Rfr-T)	0.87%	0.73%
p(Ta-T)	0.00%	0.00%
p(ST-So)	2.70%	2.61%
p(ST-D)	5.26%	5.09%
p(Rfr-D)	1.27%	1.77%
p(T-D)	0.00%	0.00%
p(Rfr-So)	0.85%	1.08%
p(T-So)	0.00%	0.00%
p(Rfr-Ta)	0.87%	0.73%
p(T-Ta)	0.00%	0.00%
p(So-D)	34.34%	30.62%
p(STa-D)	10.67%	9.14%
p(STa-Ta)	7.54%	5.65%
P(STa-So)	3.10%	3.56%
p(Ta-So)	3.56%	2.56%
p(Ta-D)	0.00%	0.00%

Table S3. Transition probabilities of cirrhotic patient with HCC treated by TACE in first-line strategy

Transition probabilities	Bondy group	SOC group
p(Ta-D)	1.69%	1.59%
p(Ta-So)	1.69%	0.35%
p(S-RF)	12.30%	3.13%
p(RF-S)	22.84%	17.92%
p(RF-T)	0.00%	0.00%
p(S-T)	0.00%	3.67%
p(T-D)	0.00%	3.55%
p(ST-D)	0.00%	1.20%
p(RF-Tar)	0.00%	0.00%
p(RF-Sor)	3.85%	0.00%
p(S-D)	12.29%	8.67%
p(So-D)	56.05%	26.52%
p(S-Tar)	15.61%	16.00%
p(Tar-So)	9.44%	4.61%
p(Tar-D)	8.63%	3.85%
p(S-So)	8.31%	6.76%

Table S4. Transition probabilities of cirrhotic patient with HCC treated by Sorafenib in first-line strategy

Transition probabilities	Bondy group	SOC group
p(So-D)	34.72%	7.14%
p(S-D)	38.47%	27.96%

A. Bondy group

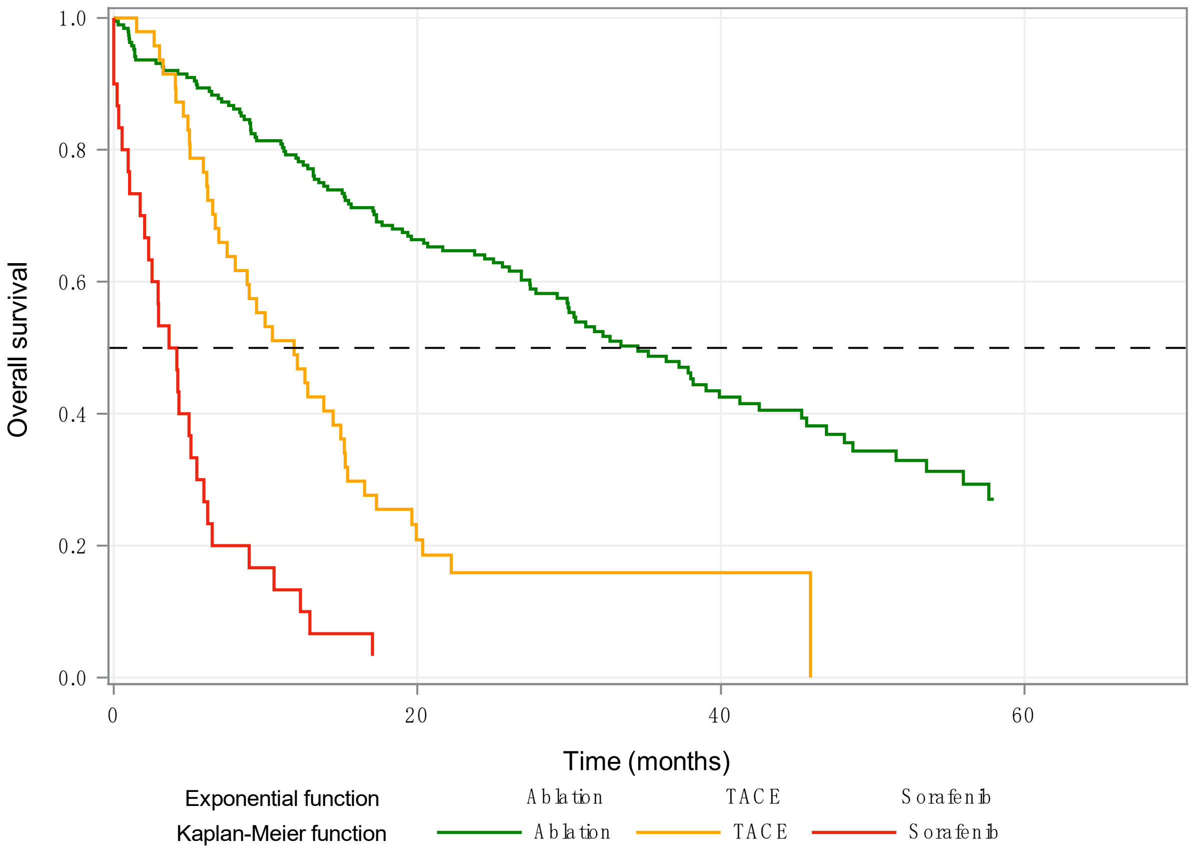

B. SOC group

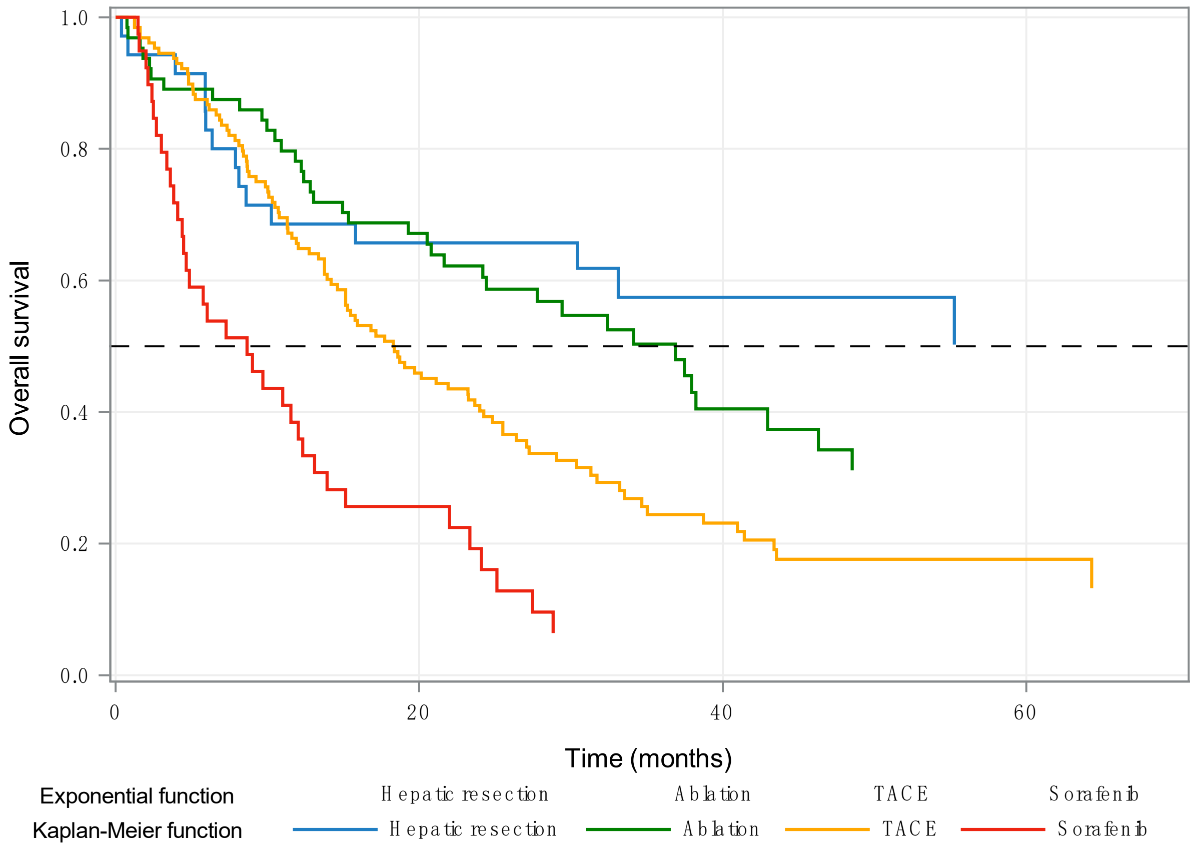

Figure S6. Model calibration by first-line treatment in the (A) Bondy and (B) SOC. Dash lines represent the Markov model results (derived from exponential function) while segmented lines represent patient-level data (Kaplan-Meier function)

Propensity Score Analysis

$P(z_i = 1|X_i)$ = b0 + b1 x age + b2 x gender + b3 x comorbidity_index + b4 x cause_of_cirrhosis + b5 x pugh_child_score + b6 x tumor_size + b7 x number_of_tumor + b8 x vascular_invasion + b9 x htp_signs + b10 x location + b11 x afp

where,

z, the probability between 0 and 1 of group assignment assumption derived from the logit model,

b0, the intercept,

bi, regression coefficients estimated by maximum likelihood,

X_i, the set of variables (age, sex, Pugh-Child score, number of tumors...).

Table S5. Characteristics of variables including in the model used for propensity score

Set of variables	Type	Values	Reference
Age	Continuous	31-92	-
Gender	Binary	Male<br>Female	Female
Comorbidity index	Ordinal	≤1<br>>1	≤1
Cause of cirrhosis	Category	Alcoholic<br>Hepatitis C<br>Hepatitis B<br>NASH<br>Others	Others
Child-Pugh score	Binary	A<br>B	B
Size of the largest tumor (mm)	Continuous	0-220	-
Number of tumor	Ordinal	Unique (=1)<br>Oligo (2-3)<br>Multi (≥4)	Multi
Vascular invasion	Binary	Present<br>Absent	Absent
HTP signs	Binary	Present<br>Absent	Absent
Location	Binary	Unilobar<br>Bilobar	Unilobar
AFP	Ordinal	≤100<br>100-1000<br>>1000ng/mL	≤100

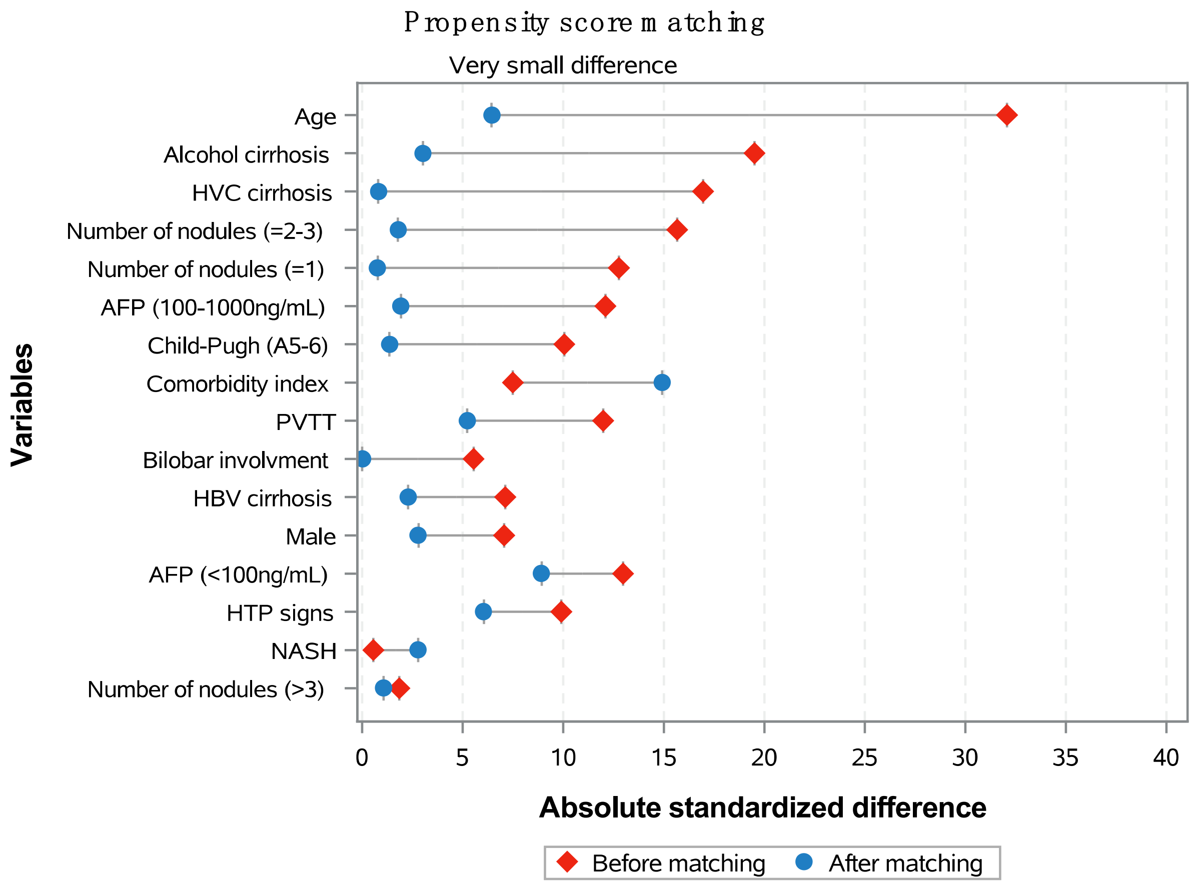

Figure S7. Balance assessment on the covariates after propensity score matching

Costs Evaluation

Table S6. Costs of in-patient HCC treatment from PMSI. Costs are valued in Euro.

In-patient HCC treatment	N (n)	Mean	SD	Minimum	Maximum	IC 95-	IC 95+
Hepatic resection	976 (976)	17 129.77 €	10 764.35 €	7 543.21 €	160 357.05 €	16 454.44 €	17 805.10 €
Ablation	1549 (1714)	3 939.35 €	5 117.75 €	413.93 €	160 357.05 €	3 684.49 €	4 194.21 €
Liver transplantation	343 (343)	51 252.89 €	22 015.90 €	21 130.20 €	176 167.11 €	48 922.95 €	53 582.83 €
TACE	2486 (3470)	3 705.61 €	3 443.79 €	404.58 €	70 480.60 €	3 570.23 €	3 840.99 €
Care after liver transplantation	16 (70)	10 528.43 €	20 273.21 €	712.16 €	95 193.01 €	594.56 €	20 462.30 €
Palliative care	89 (133)	4 343.64 €	2 474.60 €	714.56 €	20 579.54 €	3 829.52 €	4 857.76 €

N (n)=Number of patients (number of distinct treatments, patient may have received multiple procedures). SD, Standard deviation. IC 95-/+: Confidence interval 95%.

Table S7. Costs of pre-operative examinations. Costs are valued in Euro.

Pre-operative examinations	Cost per unit	Sources
Hepatologist consultation	28.00 €	AMELI
Liver ultrasonography	52.45 €	AMELI
Liver MRI	69.00 €	AMELI
Liver CT-scan	50.54 €	AMELI
Liver biopsy	196.18 €	AMELI
Nurse consultation	4.26 €	Micro-costing
Multidisciplinary meeting	91.96 €	Micro-costing
Anesthetic consultation	46.00 €	AMELI
Biology	50.83 €	Biolam

AMELI : https://www.ameli.fr/accueil-de-la-ccam/index.php; Biolam : https://www.ameli.fr/l-assurance-maladie/statistiques-et-publications/donnees-statistiques/actes-de-biologie-medicale/biolam/biolam-2013-2015.php

Table S8. Direct medical costs of out-patient HCC treatment. Costs are valued in Euro.

Out-patient treatment	Cost per unit	Sources
Cortancyl 5mg (SANOFI AVENTIS)	1.85 €	CEPS
Advagraf 5mg (ASTELLAS PHARMA)	298.28 €	CEPS
Cellcept 500mg (ROCHE)	55.73 €	CEPS
Sorafenib 200mg (BAYER)	3 261.64 €	CEPS

CEPS: Comité Economique des Produits de Santé, http://medicprix.sante.gouv.fr/medicprix.

Table S9. Costs of follow-up surveillance after curative or palliative treatment. Costs are valued in Euro.

Follow-up examinations	Cost per unit	Sources
Hepatologist consultation	28.00 €	AMELI
Liver ultrasonography	52.45 €	AMELI
Liver MRI	69.00 €	AMELI
Liver CT-scan	50.54 €	AMELI
Biology	50.83 €	Biolam

AMELI : https://www.ameli.fr/accueil-de-la-ccam/index.php; Biolam : https://www.ameli.fr/l-assurance-maladie/statistiques-et-publications/donnees-statistiques/actes-de-biologie-medicale/biolam/biolam-2013-2015.php

Table S10. Costs of in-patient follow-up (care after liver transplantation, palliative care) from PMSI. Costs are valued in Euro.

In-patient follow-up	N (n)	Mean	SD	Minimum	Maximum	IC 95-	IC 95+
Care after LT	16 (70)	10 528.43 €	20 273.21 €	712.16 €	95 193.01 €	594.56 €	20 462.30 €
Palliative care	89 (133)	4 343.64 €	2 474.60 €	714.56 €	20 579.54 €	3 829.52 €	4 857.76 €

N (n)=Number of patients (number of distinct treatments, patient may have received multiple procedures). SD, Standard deviation. IC 95-/+: Confidence interval 95%.

LT, liver transplantation.

Table S11. Direct medical costs of HCC management from French Insurance. Costs are valued in Euro.

Health states	Pre operative examinations [a]	In-patient procedures	Follow-up exams[b]	Outpatient medications[c]	Average total (3-months cycle)
Transplantation	536 €	51 253 €	-	-	51 789 €
Resection	536 €	17 130 €	-	-	17 666 €
Ablation	536 €	4 359 €	-	-	4 895 €
TACE	536 €	5 172 €	-	-	5 708 €
Sorafenib	536 €	-	-	5 870 €[d]	6 407 €
Death	-	-	-	-	0 €
Follow-up after LT	-	134 €[e]	79 €	1 301 €[f]	1 515 €
Follow-up after resection	-	-	146 €	-	146 €
Follow-up after ablation	-	-	146 €	-	146 €
Follow-up after TACE	-	232 €[g]	146 €	-	379 €

[a]Pre-operative examinations include specialist consultation in hepatology, liver ultrasound, abdominal CT-scan or MRI, liver biopsy, multidisciplinary consultation meeting, anesthesia consultation and biological exams. [b]Follow-up exams include specialist consultation, biology exams, and a CT scan or a liver MRI. [c]2.1% VTA rate was applied. [d]Compliance rate of Sorafenib was 60% of daily planned dose in SHARP trial. This rate was applied to the cost of outpatient medications. [e]In-patient procedure refers to « Care after liver transplantation » DRG. [f]Outpatients medications after liver transplantation were immunosuppressive drugs (Cortancyl®, Advagraf® and Cellcept®). [g]In-patient procedure refers to « Palliative care » DRG. TACE, trans-arterial chemoembolization; LT, liver tranplantation.

Table S12. Results of the cost-effectiveness analyses, by first-line treatment on a 5-year horizon. These results were weighted by the probabilities of each first-line treatment in the Bondy and standard of care groups and used to calculate the final ICER. The respective mixes of first-line treatments in each group used to estimate the average ICER came from the cohort distribution.

Probability of first-line treatment	Bondy group	SOC group
Hepatic resection	0.4 %	13.2 %
Ablation	70.7 %	24.0 %
TACE	17.7 %	48.1 %
Sorafenib	11.2 %	14.7 %

First-line	Costs and life-years per patient	Bondy group	SOC group	Net effects	ICER (€/LYG)
Hepatic resection	Cost	€101 ($111)	€3,513 ($3,864)	€-3,412 ($-3,753)	
	Life-years	0.1	1.7	-2.5	
Ablation	Cost	€12,187 ($13,406)	€4,101 ($4,511)	€8,086 ($8,895)	
	Life-years	7.7	2.7	7.3	
TACE	Cost	€2,266 ($2,493)	€12,098 ($13,308)	€-9,832 ($-10,815)	
	Life-years	0.7	3.5	-3.6	
Sorafenib	Cost	€1,322 ($1,454)	€2,694 ($2,963)	€-1,372 ($-1,509)	
	Life-years	0.2	0.5	-0.3	
Average total	Cost	€15,876 ($17,464)	€22,406 ($24,647)	€-6,530 ($-7,183)	**Dominant**
	Life-years	8.7	8.4	0.3	

Costs and life years are expressed as average for a patient and were discounted by 3%. The net effect could be a reduction in costs (negative values) or an increase in costs (positive values).

TACE, trans-arterial chemoembolization; ICER, incremental cost-effectiveness ratio; LYG, life-years gained.

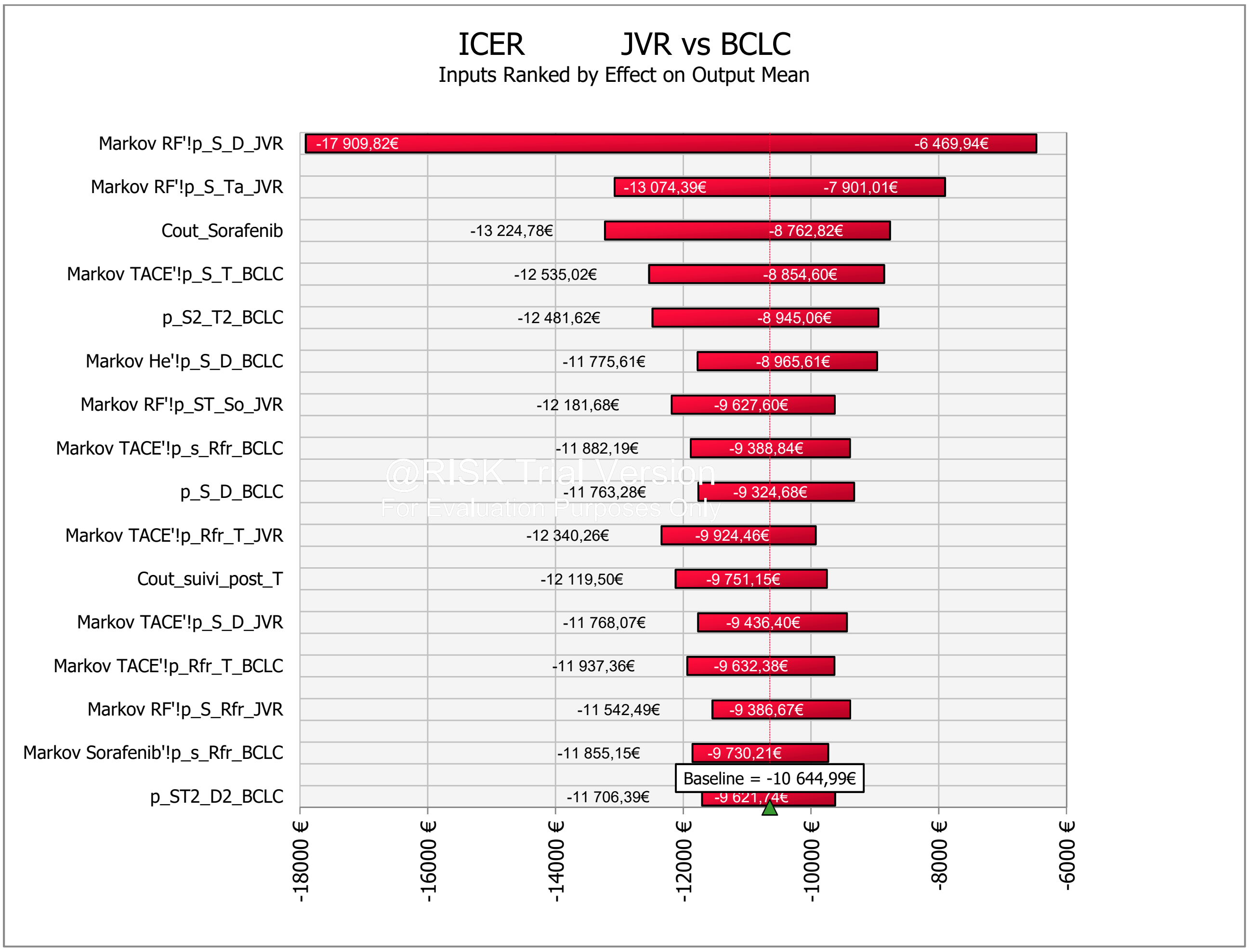

@RISK Trial Version
For Evaluation Purposes Only

Figure S8. Results of the deterministic univariate analysis.
